# The impact of an intervention to increase uptake to structured self-management education for people with type 2 diabetes mellitus in primary care (the embedding package), compared to usual care, on glycaemic control: study protocol for a mixed methods study incorporating a wait-list cluster randomised controlled trial

**DOI:** 10.1186/s12875-019-1038-0

**Published:** 2019-11-07

**Authors:** Melanie J. Davies, Caroline A. Kristunas, Abualbishr Alshreef, Simon Dixon, Helen Eborall, Agnieszka Glab, Lisa Huddlestone, Nicky Hudson, Kamlesh Khunti, Graham Martin, Alison Northern, Mike Patterson, Rebecca Pritchard, Sally Schreder, Bernie Stribling, Jessica Turner, Laura J. Gray

**Affiliations:** 1 Diabetes Research Centre, University of Leicester, Leicester, UK; 2 Leicester Diabetes Centre, University Hospitals of Leicester NHS Trust, Leicester, UK; 3 Biostatistics Research Group, Department of Health Sciences, College of Life Sciences, University of Leicester, George Davies Centre , University Road, Leicester, LE1 7RH UK; 4 School of Health and Related Research, University of Sheffield, Sheffield, UK; 5 Centre for Reproduction Research, School of Applied Social Science, De Montfort University, Leicester, UK; 6 THIS Institute, University of Cambridge, Cambridge, UK

**Keywords:** Type 2 diabetes, Self-management, Structured education, Diabetes self-management, Diabetes education, Randomised controlled trial, Wait-list, Cluster randomised trial

## Abstract

**Background:**

Approximately 425 million people globally have diabetes, with ~ 90% of these having Type 2 Diabetes Mellitus (T2DM). This is a condition that leads to a poor quality of life and increased risk of serious health complications. Structured self-management education (SSME) has been shown to be effective in improving glycaemic control and patient related outcome measures and to be cost-effective. However, despite the demonstrated benefits, attendance at SSME remains low. An intervention has been developed to embed SSME called the ‘Embedding Package’. The intervention aims to address barriers and enhance enablers to uptake of SSME at patient, healthcare professional and organisational levels. It comprises a marketing strategy, user friendly and effective referral pathways, new roles to champion SSME and a toolkit of resources.

**Methods:**

A mixed methods study incorporating a wait-list cluster randomised trial and ethnographic study, including 66 UK general practices, will be conducted with two intervention start times (at 0 and 9 months), each followed by an active delivery phase. At 18 months, the intervention will cease to be actively delivered and a 12 month observational follow-up phase will begin. The intervention, the Embedding Package, aims to increase SSME uptake and subsequent improvements in health outcomes, through a clear marketing strategy, user friendly and effective referral pathways, a local clinical champion and an ‘Embedder’ and a toolkit of resources for patients, healthcare professionals and other key stakeholders.

The primary aim is, through increasing uptake to and attendance at SSME, to reduce HbA1c in people with T2DM compared with usual care. Secondary objectives include: assessing whether there is an increase in referral to and uptake of SSME and improvements in biomedical and psychosocial outcomes; an assessment of the sustainability of the Embedding Package; contextualising the process of implementation, sustainability of change and the ‘fit’ of the Embedding Package; and an assessment of the cost-effectiveness of the Embedding Package.

**Discussion:**

This study will assess the effectiveness, cost-effectiveness and sustainability of the Embedding Package, an intervention which aims to improve biomedical and psychosocial outcomes of people with T2DM, through increased referral to and uptake of SSME.

**Trial registration:**

International Standard Randomised Controlled Trials Number ISRCTN23474120. Assigned 05/04/2018. The study was prospectively registered. On submission of this manuscript practice recruitment is complete, participant recruitment is ongoing and expected to be completed by the end of 2019.

## Background

Diabetes affects approximately 425 million people globally [1], with approximately 3.8 million in the United Kingdom (UK) [2], a figure that continues to rise [3]. It is estimated that by 2035, diabetes will account for 17% of National Health Service (NHS) expenditure [4]. Around 90% of people with diabetes have Type 2 Diabetes Mellitus (T2DM) [1], a serious, progressive, chronic disease, which leads to poor quality of life and increased prevalence of costly long term health complications. Despite advances in pharmacological interventions, management of T2DM remains a challenge.

Structured self-management education (SSME) for T2DM has been shown to be both beneficial and cost-effective [5–8]. Programmes such as DESMOND [6, 7], X-PERT [8] and the Diabetes Manual [9–11], have shown SSME to be associated with improved biomedical (e.g. HbA1c, lipids, weight, blood pressure), psychosocial (e.g. depression, quality of life, hypoglycaemia rates), behavioural, and medical outcomes [6–8, 12, 13]. A recent systematic review and network meta-analysis found that SSME on averaged reduced HbA1c by over 0.4%, with the greatest benefits seen in those with poor glycaemic control (HbA1c > 7.0%), aged less than 65 years and non-white participants [14]. Powerfully, another systematic review combining data from 42 trials, found a 26% reduced risk of all cause morality in those who had attended diabetes SSME compared to standard care [15].

Unfortunately, despite the increase in the quality and quantity of the evidence base since the National Institute for Clinical Excellence (NICE) first recommended SSME programmes and made SSME for T2DM a national priority in the UK [16], rates of uptake to SSME for those with T2DM have remained persistently low. The recent addition of a Quality and Outcomes Framework (QOF) indicator for referral to SSME in those with newly diagnosed T2DM [17] has improved the rate at which education is offered to people with newly diagnosed T2DM, with 74.5% of those with newly diagnosed diabetes (type 2 and other) being offered SSME within 12 months of diagnosis in 2016 as opposed to 47.4% in 2013 [18]. However, referral does not equate to uptake; moreover, many of these programmes may not be evidence based or meet the NICE criteria. The most recent national figures (2016) show that only 8.3% of T2DM patients were recorded as having attended SSME within 12 months of diagnosis [18].

Evidence suggests that poor participation is due to multiple patient, healthcare professional (HCP) and organisational factors. At the level of HCPs, barriers include: insufficient investment, insufficiently trained educators, lack of staff capacity, absence of public health marketing for diabetes awareness, lack of integration into patient pathways, poor IT systems for tracking the patient, absence of an infrastructure for organisation-wide education, HCPs not advocating or recognising the positive outcomes of self-management education, the misperception that education is expensive, and lack of consideration of patient access issues [19]. A review conducted in 2017 highlighted a number of commonly reported patient barriers to access SSME, which included: issues with timing and length of courses; access/transportation issues; family/work commitments; lack of information and the benefits of attending not being communicated by the HCP; patients feeling happy with the information they had already been provided with (and so seeing SSME attendance as unnecessary) [20].

An intervention titled the ‘Embedding Package’ has been developed in order to increase uptake to SSME by people with T2DM in primary care, with the overall intention of improving glycaemic control. The Embedding Package was designed to address barriers and enhance enablers to uptake at patient, HCP, and organisation levels, including SSME providers and commissioning bodies. This package has been piloted and refined in a feasibility study (Davies et al. Increasing uptake of self-management education programmes for type 2 diabetes in a multi-ethnic primary care setting: A feasibility study. 2019. In preparation). The mixed methods study outlined in this protocol is designed to assess the effectiveness, cost effectiveness, and sustainability of this Embedding Package in comparison with usual care using a wait-list cluster randomised controlled trial (RCT) design with an integrated ethnographic study.

## Methods/design

### Objectives

The primary objective of this ambitious mixed methods study is to assess whether the Embedding Package, by increasing uptake and attendance at structured education, reduces HbA1c in people with T2DM compared with usual care. This objective will be addressed through a wait-list cluster RCT.

The secondary objectives are to:

- assess whether the Embedding Package increases referral to and uptake of structured education, as well as improving biomedical and psychosocial outcomes;
- assess sustainability of the Embedding Package using an observational follow-up period;
- contextualise the process of implementation, sustainability of the change and the ‘fit’ of the Embedding Package within routine practice;
- assess cost-effectiveness of the Embedding Package.

### Summary of study design

This 30 month open-label trial is testing a complex intervention, the Embedding Package, compared to usual care (see Table 1 for World Health Organization Trial Registration Data Set). The intervention is complex with elements delivered at multiple levels (practice, provider and Clinical Commissioning Group (CCG)). The study has been designed in line with best practice guidelines to seek to provide a comprehensive understanding of whether and how the intervention works, as well as providing cost data to inform its potential roll-out in the future [21]. Accordingly, the study comprises an 18 month wait-list cluster RCT, similar to a stepped wedge design but with a single step during the study, to ascertain the effectiveness and cost-effectiveness of the intervention, followed by a 12 month observation to ascertain whether changes are maintained after study support is withdrawn. This is a mixed methods project and to better understand and evaluate the process of implementation, ethnographic work will run alongside the trial.

**Table 1:** World Health Organization Trial Registration Data Set

Item	
Primary Registry and Trial Identifying Number	International Standard Randomised Controlled Trial Number, ISRCTN23474120
Date of Registration in Primary Registry	05/04/2018
Secondary Identifying Numbers	NA
Source(s) of Monetary or Material Support	This project is funded by the National Institute for Health Research (NIHR) Programme Grants for Applied Research (Increasing uptake of effective self-management education programmes for type 2 diabetes in multi-ethnic primary care settings RP-PG-1212-20,004).
Primary Sponsor	University of Leicester uolsponsor@le.ac.uk
Secondary Sponsor(s)	NA
Contact for Public Queries	Professor Melanie Davies melanie.davies@uhl-tr.nhs.uk Principal Investigator Diabetes Research Centre, University of Leicester, Leicester, UK.
Contact for Scientific Queries	Professor Melanie Davies melanie.davies@uhl-tr.nhs.uk Principal Investigator Diabetes Research Centre, University of Leicester, Leicester, UK.
Public Title	Evaluating the impact of increasing uptake of self-management education programmes for Type 2 Diabetes in primary care: A wait-list cluster randomised controlled trial
Scientific Title	Evaluating the impact of increasing uptake of self-management education programmes for Type 2 Diabetes in primary care: A wait-list cluster randomised controlled trial
Countries of Recruitment	UK
Health Condition(s) or Problem(s) Studied	Type 2 Diabetes
Intervention(s)	Intervention – Embedding Package It comprises four key components: 1. clear marketing strategy for SSME; 2. user friendly and effective referral pathways; 3. new/amended roles including a local clinical champion and an ‘Embedder’; 4. toolkit of resources (for patients, HCPs and other key stakeholders). Control – Usual Care Eeach practice will continue to provide their usual activities related to SSME whilst in the control period.
Key Inclusion and Exclusion Criteria	Patient Inclusion criteria: registered at a participating practice; aged ≥18 years old; coded in their primary care medical record as diagnosed with T2DM before or during the step (to be re-assessed at each data extraction point). Patient exclusion criteria: coded in their primary care medical records as having a terminal illness, housebound or in residential care; a dissent code in their primary care medical records for researcher to access clinical data. See eligibility section for full inclusion/exclusion for all parts of the study.
Study Type	Type of study - interventional Method of allocation – cluster randomised Masking – none Assignment – wait list study, practices randomised 1:1 to immediate intervention or to wait Purpose – improve outcomes
Date of First Enrolment	First practice 06/08/2018
Target sample size	66 practices 2050 participant questionnaires
Recruitment status	As of 02/10/19 66 practices recruited, 1920 questionnaires returned
Primary outcome(s)	HbA1c
Key secondary outcomes	Referral and uptake rates to SSME

#### RCT design (months 0–18)

**Fig. 1:**
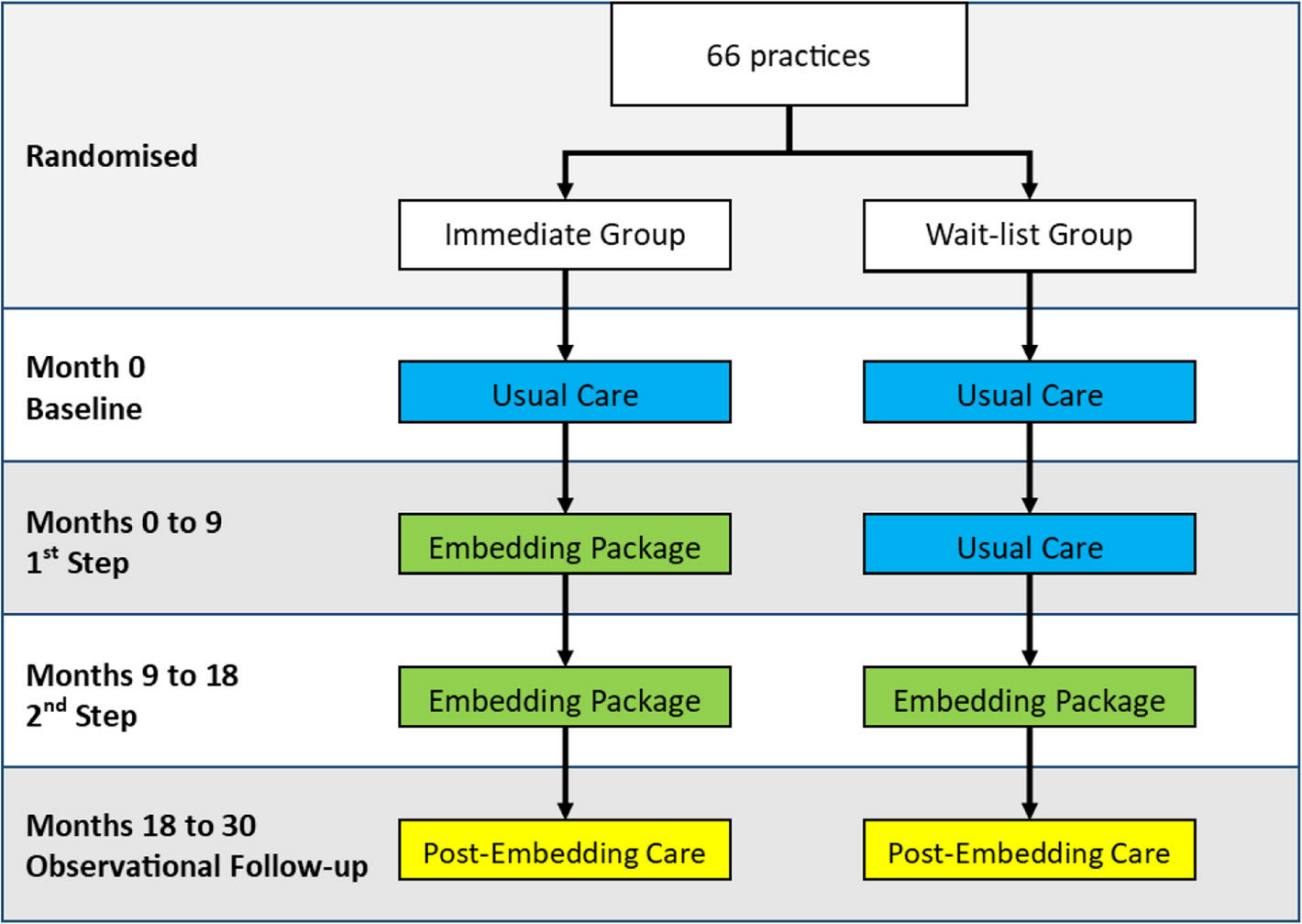
Outline of the design of the trial

Baseline data have been collected at month 0. Practices will be randomised 1:1 to 1) the immediate group who receive the Embedding Package from months 0 to 18, or 2) the wait-list group who provide usual care for months 0–9 and receive the Embedding Package for months 9 to 18 (Fig. 1). Since data are collected from each step, each practice acts as its own control (immediate group uses the baseline data as its control, the wait-list group uses data collected between 0 and 9 months). To limit potential contamination, for the aspects of the intervention that are targeted at CCG/locality/provider level (e.g. social marketing initiatives), we will request that as far as possible, these are first targeted at practices participating in the Embedding Package, and only aimed at wait-list practices when these have crossed over to receive the intervention.

#### Observational follow-up design (months 18–30)

The 12-month observational follow-up is designed to investigate whether any improvements observed during the RCT are maintained. During this time the study team will no longer actively reinforce the Embedding Package, but practices can continue using the intervention provided during the RCT, if they choose to do so.

#### Integrated ethnographic study (months 0–30)

The integrated ethnographic study is designed to provide comprehensive data on the process of implementation and the fit of the intervention. Qualitative data will be gathered from observations and semi-structured interviews. The following will be undertaken:

1. Use the formative findings from the immediate group to refine, tailor and enhance the Embedding Package and its implementation in the second step.
2. Use the data to provide additional evidence about the context of implementation and sustainability of change in primary care
3. Investigate the degree to which active work to embed SSME has continued in the observational follow-up period
4. Examine the extent to which changes are perceived to have been sustained
5. Identify any changes in the stakeholder organisations with a bearing on sustainability.

Cost estimates will also be generated from structured interviews with staff from SSME providers. The interview and observational data collection tools were developed and tested in the preceding feasibility study (Davies et al. Increasing uptake of self-management education programmes for type 2 diabetes in a multi-ethnic primary care setting: A feasibility study. 2019. In preparation).

### Primary and secondary outcome measures

All outcomes will be measured at baseline (0 month), first (0–9 months), and second (9–18 months) steps, as well as over the observational follow-up (18–30 months), except for the self-report data which will only be measured once by questionnaire (during the first step). All biomedical patient level outcomes will be extracted from primary care electronic medical records. For most of these outcomes, the last measurement within that time period will be used. For example, for the first step HbA1c will be defined as the last HbA1c measurement between months 0 and 9. If the variable has not been measured over that time period then it will be deemed missing.

#### Primary outcome

The primary outcome is patient-level HbA1c compared between the control and intervention conditions in the RCT i.e. the baseline measure in the immediate group, and baseline and first step in wait-list group compared with first and second steps in immediate group and second step in wait-list group.

#### Secondary outcomes

The secondary biomedical measures to be extracted from primary care at the patient level are: Body Mass Index (BMI); weight; total, Low-Density Lipoprotein (LDL) and High-Density Lipoprotein (HDL) cholesterol; systolic and diastolic blood pressure; glucose, blood pressure and lipid lowering medications; smoking status; hospital admissions; cardiovascular risk score.

Process outcomes will reflect how the intervention is implemented and aid understanding of effectiveness. They will be measured at the patient, practice, or provider level as appropriate, and will include the following: whether or not the patient was referred to and attended SSME (main secondary outcome); type of SSME; percentage of eligible individuals referred to education; percentage of eligible individuals who attended education; percentage of eligible individuals who declined education; number, timing and venue of available education sessions; number of trained educators.

Secondary psychosocial and process outcomes will also be self-reported by a subset of patients at a single time-point during the first step so that psychosocial outcomes can be compared between those in the intervention and control arms (it is estimated around 2000 questionnaires will be returned). This data will be collected by questionnaire; all participants will be sent a short questionnaire they can further opt to complete an additional questionnaire. The self-reported outcomes from the short questionnaire are: whether or not the patient was referred to and attended SSME; and where the patient has previously received diabetes information from. Those completing the additional questionnaire will provide data on patient activation measure; well-being (W-BQ12); Problem Areas in Diabetes (PAID) score.

Additionally, back end website data on the extent of user engagement (e.g. length of time for which individual pages were viewed and the number of occasions etc.) with specific tools in the online toolkit will be collected.

### Trial participants

#### Recruitment

Before patients were contacted CCGs and their associated SSME providers were approached regarding the study. They needed to support the study by implementing the aspects of the Embedding Package that are aimed at providers and commissioners themselves. Participating providers will contribute data on activities relating to the Embedding Package for use in the cost-effectiveness analysis, on the availability of education sessions and on the number of educators. In CCGs where the provider declined participation, Embedders will work solely and directly with the practices.

Eligible practices within these localities were recruited. Practices with an interest in taking part will contact the research team to discuss their possible participation. Patient-level data will be extracted from participating practices computer systems. Cost-effectiveness data will be collected through the recording of Embedding Package activity. All patients diagnosed with T2DM at the start of each step (including those diagnosed during the step), registered at the practice, and meeting the eligibility criteria will have their pseudonymised data extracted for the outcome measures and will be invited to complete a consent form to link extracted data with self-reported information.

Sampling to the integrated ethnographic study will be purposive, and based on practice characteristics (location, type of setting - rural, suburban or urban, practice size and socio-economic factors), provider characteristics (structured education programme) and CCG characteristics (sustainability and transformation plans) [22]. Adoption of this selection criteria will enable the identification of a varied sample of practices with a view to developing an in-depth explanation of the extent of success of implementing the Embedding Package at different levels and in different settings, and inform how to optimise implementation of the package (and similar initiatives) in different contexts. In the first step, this will be informed by demographic profile and discussions with the trial co-ordinators, and will aim to generate a maximum variation sample [23]. Sampling during the second step will be theoretically informed by mid-term progress data to explore the challenges involved in implementing and sustaining the Embedding Package in a variety of circumstances. Data will also be collected from organisations associated with the delivery of the Embedding Package in these practices, including CCGs, providers, NHS England regional offices, area teams, and commissioning support units. Practices and associated organisations sampled in the first step will continue to be included in data collection in the second step.

A sub-sample of practices and all of the participating providers will also provide more detailed information on the costs of the individual activities through interviews with a designated staff member (such as the SSME service manager).

Practice recruitment is complete. The first practice was randomised on 06/August/2018, the last practice was randomised on 22/February/2019. Data collection will be completed by 22/August/2021. Participant recruitment started in August 2018 and is expected to be completed by December 2019.

#### Eligibility criteria

Practices, patients and stakeholders will be recruited and take part in relevant study activities if they meet all of the relevant criteria, as detailed below.

#### Practices

Practice should be located within a participating CCG; use either EMIS Web or TPP SystmOne (required for data extraction); able to refer people with T2DM to a structured education programme which meets NICE criteria; willing to sign a data sharing and data collection agreement with PRIMIS allowing the collection of pseudonymised patient data and, where patient consent is given, identifiable data, as required for analysis; where appropriate willing to have a sample of meetings and consultations observed or to be interviewed.

#### Patients – data extraction and mail-out

All patients registered at a participating practice and meeting the following eligibility criteria will have pseudonymised data extracted. Eligibility will be assessed at each step and therefore the study is of an open cohort design. If included patients die, or leave the practice, they will be included up until this point. All eligible patients at baseline will be invited to join the self-report component and consent to link data.

Patient inclusion criteria: registered at a participating practice; aged ≥18 years old; coded in their primary care medical record as diagnosed with T2DM before or during the step (to be re-assessed at each data extraction point); willing and able to provide informed consent (applicable to optional consent form and questionnaire booklet only); able to understand written English to a level sufficient to enable an understanding of the research and their participation within it (applicable to optional consent form and questionnaire booklet only). Patient exclusion criteria: coded in their primary care medical records as having a terminal illness, housebound or in residential care; a dissent code in their primary care medical records for researcher to access clinical data.

#### Patients – integrated ethnographic study

Patients who express an interest to participate in the ethnographic study and meeting the following criteria will be eligible for the ethnographic study. Patient inclusion criteria: meet all of the patient eligibility criteria for the trial; is able to attend the practice unaided or with a carer or support (applicable to observations of consultations only). Patient exclusion criteria: unable to understand spoken English to a level sufficient to enable an understanding of the research and their participation within it.

#### Stakeholders

Stakeholders are individuals who work at participating practices, members of CCGs, education providers, or in attendance at meetings in a patient and public involvement (PPI) capacity, who may be approached to participate in a number of activities. Stakeholders will be eligible for inclusion in the integrated ethnographic study if they meet all of the following inclusion/exclusion criteria. Stakeholder inclusion criteria: employed by a participating practice/CCG/provider organisation, or involved in the delivery or commissioning of any aspect of the Embedding Package in a participating practice/CCG/provider organisation; willing and able to give informed consent (written or verbal). Stakeholder exclusion criteria: unable to understand written and spoken English to a level sufficient to enable an understanding of the research and their participation within it.

### Study procedures

#### Informed consent

Informed consent will not be required for the data extraction element of the study as patients will not be directly approached and their data will be extracted pseudonymously. However, the Caldicott Guardian of each participating practice will be required to consent to the extraction. A summary of the method of consent for each study element is provided in Table 2.

**Table 2:** Summary of method of consent per study element

Study Element	Method of Consent
Complete questionnaire booklet and link with routine clinical data (patients)	Written via mail-out
Interviews - Face-to-Face	Written
Interviews - Telephone	Verbal
Observations - SSME Session	Written
Observations – Consultations/Meetings	Verbal

All members of the research team receiving informed consent will be Good Clinical Practice certificated, and authorised to do so by the Chief Investigator. Original signed consent forms will be retained and participants will be given or sent a copy. The PIS will detail the exact nature of the study, the implications, and any risks involved in taking part.

#### Consent to complete questionnaire booklet and link with routine clinical data

Patients will be approached by postal invitation sent from the practice along with information about the study and a questionnaire booklet containing a short questionnaire and consent form. Patients will also be asked to provide consent for the research team to link their responses to the data extracted from GP practices and their record held by the local SSME provider, if such a record exists. Patients will be able to decline participation, or to indicate willingness to participate in either the self-report and data linkage (participation in one of these does not necessitate participation in any other activity). Patients could also opt to complete an additional questionnaire which included patient reported outcome measures.

#### Consent to interviews

Informed consent will be received by a member of the research team when a patient or stakeholder agrees to participate in an interview. All participants will have the opportunity to discuss the purpose of the interview and the PIS, ask any questions they have, and then to decide whether they will participate. For face-to-face interviews, the interviewer will obtain written informed consent immediately prior to the interview. For telephone interviews, the interviewer will audio record the reading out of the latest approved version of the consent form and the participant’s agreement with clauses, the consent form will then be annotated with the recording identification code, signed, and dated by the interviewer. The original copy of the consent form will be retained in the study file and a copy will be provided, either at the time of the interview or by post, to the participant.

#### Consent to observation of SSME sessions

The educator(s) of a sample of one-to-one or group-based SSME sessions will be given a copy of the stakeholder PIS and will have the opportunity to consider this information and ask any questions. They will then be asked to provide written consent and confirm the dates of the sessions they would be happy to have observed. If the educator(s) does not consent then no participants will be approached. After educator consent has been received, when booking participants onto one of these sessions, the SSME administrator will inform each participant verbally about the study and the presence of an observer. If a participant does not want to attend a session that is being observed, they will be booked onto a session on an alternative date. Participants who give verbal consent (when booking) to be observed will be sent a PIS. Immediately prior to the session, a verbal delivery of the PIS will be given by the researcher. Prior to requesting written consent, potential participants will be given the opportunity to have any questions related to the study or their participation within it addressed.

#### Consent to observation of healthcare consultations

The ethnographic team will attend clinics in order to observe consultations where discussions about SSME may take place between patients and HCPs. It will not be practical or appropriate to obtain written consent from the HCP or the patient on the day, as this would present a burden to the practice and introduce delays in the clinic/appointment schedule. Therefore, verbal permission will be obtained from the patient and the HCP ensuring that those who wish to opt out can easily make this known. Written and verbal versions of the PIS will be provided and all participants will be given time to consider the information and ask questions. Patients will be informed of the possible presence of an observer by the practice receptionist when they arrive for their appointment. It will be made clear that they are free to ask that their consultation is not observed. The observer will offer to withdraw without any reason having to be given by either the patient or the HCP, and in any event will withdraw if there is any doubt about the appropriateness of their presence.

#### Consent to observation of practice/provider meetings

A sample of meetings where SSME is discussed may be observed. Verbal consent will be sought from the appropriate person in the organisation and/or the Chair of the meeting. When possible, those due to attend the meeting will be informed about the study by the observer prior to the meeting, by providing them with a copy of the stakeholder PIS. If the Chair is willing, at the start of the meeting the observer will explain their role, that anonymity is guaranteed and that they will absent themselves at any time if anyone would rather they were not there; they will then receive verbal consent from all those present. If any individual does not wish to give consent, the observer will withdraw from the meeting. Participants may also request that the observer withdraws temporarily, for example if part of the meeting relates to issues that are confidential, or are not pertinent to the focus of the study. The researcher will respect all such requests.

#### Data collection

Provider-level process outcome data will be collected from the provider using a brief questionnaire at months 0, 9, 18, and 30. Patient-level biomedical and process outcome data will be collected through pseudonymous extraction from primary care data using Read codes. These data will be aggregated where appropriate to calculate the practice-level process outcomes. Data will be extracted at three time-points (month 0, month 9 and 18 collected together, and month 30).

Data extraction procedures are based on those used previously in similar studies [24], and the preceding feasibility study (Davies et al. Increasing uptake of self-management education programmes for type 2 diabetes in a multi-ethnic primary care setting: A feasibility study. 2019. In preparation). Practices can extract their own data, pseudonymise it and then transfer it electronically to the study team at the University of Leicester, or this can be done remotely by PRIMIS. Data extraction will be performed using MIQUEST software. The only key identifier to be extracted will be NHS numbers which will be encrypted into a unique hash (#) code by an Open-Pseudonymiser and CHART software and stored in a spreadsheet containing only pseudonymised data. This will be appropriately and securely transferred to the research team at University of Leicester. PRIMIS will not be able to identify patients at any point during the study. The practice ID code will not be encrypted as it will be required by the study team to identify which randomisation arm the data belong in.

The transferred spreadsheet will contain one line per patient pseudonymised data from all people with T2DM registered at the participating practices during each step (See Table 3). Data recorded over the measurement periods (months 0, 0–9, 9–18, and 18–30) will be extracted.

**Table 3:** Data extracted from primary care at each time point

Variable	Value of Interest	To be extracted
Type 2 diabetes diagnosis	First recorded	Value and date
NHS number	Last recorded	Pseudonymised value
Age	Last recorded	Value
Sex	Last recorded	Value
Ethnicity	Last recorded	Value
Smoking status	Last recorded	Value and date
SSME: Referred	Last recorded	Value and date
SSME: Not suitable	Last recorded	Value and date
SSME: Declined	Last recorded	Value and date
SSME: Did not attend	Last recorded	Value and date
SSME: Not completed	Last recorded	Value and date
SSME: Attended	Last recorded	Value and date
SSME: Completed	Last recorded	Value and date
HbA1c	Last recorded within measurement period	Value and date
Body mass index	Last recorded within measurement period	Value and date
Weight	Last recorded within measurement period	Value and date
Height	Last recorded	Value and date
Total cholesterol	Last recorded within measurement period	Value and date
LDL cholesterol	Last recorded within measurement period	Value and date
HDL cholesterol	Last recorded within measurement period	Value and date
Systolic blood pressure	Last recorded within measurement period	Value and date
Diastolic blood pressure	Last recorded within measurement period	Value and date
Cardiovascular risk score	Last recorded within measurement period	Value and date
Medication: Glucose lowering	All recorded within measurement period	Value and date
Medication: Lipid lowering	All recorded within measurement period	Value and date
Medication: Blood pressure lowering	All recorded within measurement period	Value and date
Hospital admission	All recorded within measurement period	Value and date

**Fig. 2:**
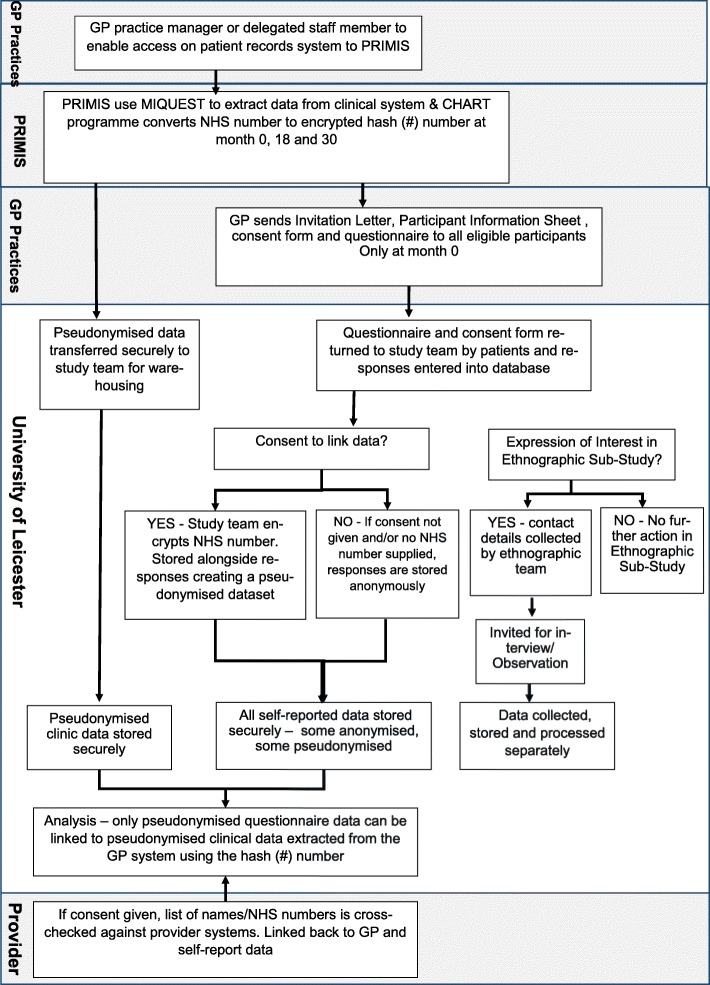
The flow of patient data

Each questionnaire booklet sent to patients via a practice mail shot will be pre-assigned a unique patient identifier. Consent and patient details (NHS number, patient name and contact details) will be collected on a separate form and will be stored separately. If the questionnaire booklet is completed and returned with consent, the NHS number will be used to identify the individual patient data in the data extracted from primary care. PRIMIS will provide the study team with the encryption algorithm so a NHS number can be converted to the unique hash (#) code to allow this linkage. Additionally, if consent is given, the list of names and NHS numbers of patients will be cross-checked against relevant provider systems, and data on SSME invitation and attendance held by the provider for these individuals will be linked with their GP and self-report data. The study team will only have access to the name and NHS number of patients returning their questionnaire booklets and will only link data of consenting patients. Secure NHSmail service will be used. Patient NHS numbers will be sent between NHSmail and NHSmail addresses. In addition, list of patients’ NHS numbers will be password protected and passwords will be sent in a separate email. The flow of patient data is shown in Fig. 2.

#### Integrated ethnographic study

Ethnographic data collection is likely to include observations of: ‘usual care’; implementation of the Embedding Package; and observations of the operationalisation of the Embedding Packing in a variety of local contexts. This will include informal discussions with healthcare, commissioning, and administration staff, the collection of key documents, including publically available information, and structured field notes, [25]; semi-structured interviews (involving stakeholders involved in commissioning, training and implementing SSME and/or the Embedding Package, and people with T2DM). Interviews will explore perceptions and experiences related to the various elements of the Package, and preferred modalities of SSME (such as group-based, one-to-one or online). Interviews will last approximately 30–45 min and may be conducted at their place of work, home, another convenient location, or by telephone, depending on participant preference. All interviews will be audio-recorded (with participant consent) and transcribed.

#### Cost-effectiveness data collection

The ‘Embedder(s)’ (i.e. the person(s) responsible for driving the implementation of the Embedding Package) will complete a simple tick-box tracker of the pre-identified implementation activities for months 0–9, 9–18 and 18–30. This tracker will cover the type of activity, the duration over which it was applied, and whether it is still ongoing, providing a census of what activities have been attempted. In addition, the tracker data will provide a measure of resource use against which unit costs can be applied to estimate the costs associated with the Embedding Package. The unit costs will be generated by structured interviews undertaken with designated staff at provider organisations within a sub-sample of practices. The interview will ask for details of staff time, consumables, and other costs that have been devoted to each individual activity over the duration that the activity was undertaken. A pro-forma for the activity data requirements will be sent in advance of the interview. A follow-up e-mail to confirm the data discussed at the meeting will be sent to the interviewees. A maximum of two further e-mails will be sent to resolve any outstanding data queries. Resource use and costs data for developing the intervention, the Embedding Package, will be obtained from the study team.

### Intervention

The Embedding Package underwent development based on a range of qualitative and experiential work, as well as piloting in an earlier feasibility (Davies et al. Increasing uptake of self-management education programmes for type 2 diabetes in a multi-ethnic primary care setting: A feasibility study. 2019. In preparation). It comprises four key components: 1. clear marketing strategy for SSME; 2. user friendly and effective referral pathways; 3. new/amended roles including a local clinical champion and an ‘Embedder’; 4. toolkit of resources (for patients, HCPs and other key stakeholders). The toolkit contains a wide selection of patient-facing resources (e.g. promotional posters, invitation letters and self-referral forms), HCP-oriented resources and guidance (e.g. document templates, guidance for recruiting staff, referring patients and increasing staff engagement) and coordination/provider/commissioner-oriented resources (e.g. audit collection and reporting, electronic administration and referral systems, and sample referral pathways). It will also include guidance on constructing and carrying out marketing and communication strategies, how to carry out local needs assessments, as well as detail about how to ensure patient accessibility and course tailoring. The new/amended roles will include the appointment of an ‘Embedder’ working across each site (or potentially up skilling of an individual already holding an analogous post) who will liaise between all relevant stakeholders to promote SSME, use of the Toolkit, communication and referrals etc. A local clinical champion in each CCG (for example the Diabetes Lead at one of the participating practices) will be identified to promote SSME across the whole locality. Together the two roles and the online Toolkit make up the ‘Embedding Package’ (the intervention).

Patients will also be able to access online versions of SSME as a complement to attendance at the group-based version as some patients may prefer education delivered via a different modality. In order to track the use of this, practice-specific log-ins will be generated that participating practices can give to patients. There will be posters in participating practices to make patients aware of this option.

To allow for any changes to the landscape of the NHS during the project, whilst maintaining the integrity of the study, any changes to the Embedding Package will only be made at Month 9, i.e. when the wait-list group begin receiving the intervention, so that the Embedding Package received during any one time period is consistent. The details of the Embedding Package that is actually delivered in each time period will be recorded and considered in secondary analyses, as appropriate.

On commencement of the study in a CCG, the Embedder will hold a Toolkit Action Plan meeting to look at which elements of the Toolkit can be implemented. This will then be written up and circulated for finalisation including assigning of tasks to relevant personnel. Review meetings will be scheduled to look at progress. Actions relating to practices will then be disseminated by the ‘Embedder’ to the relevant staff within each practice, and additional meetings arranged, if necessary.

### Control

Usual care will be practice-dependent; therefore, each practice will continue to provide their usual activities related to SSME whilst in the control period. These activities vary greatly between CCGs and their associated practices, and due to the ever-changing landscape of the NHS, usual care may evolve over time. However, usual care will be monitored and recorded within all practices.

### Randomisation

This is an open-label trial as it will not be possible to blind practices to their treatment arm. Practices will be randomised prior to baseline (month 0) in a 1:1 fashion to either: Immediate group (receive the Embedding Package for months 0–18), or wait-list group (provide usual care for months 0–9 then receive the Embedding Package for months 9–18). Randomisation will be stratified by CCG, and generated and implemented by a statistician. The statistician will provide the study team with the randomisation outcome so that they can inform practices of their allocation.

### Analysis methods

#### RCT

Data from the RCT will be analysed once data collection is complete. Descriptive summary statistics of baseline characteristics and process variables (e.g. number of education sessions available, back end data for the Toolkit and MyDesmond websites, usual care delivery, etc.) will be produced, using mean (standard deviation) for normally-distributed variables, median (interquartile range) for non-normally distributed variables, and count (percentage) for categorical variables.

The primary analysis will compare HbA1c between the control and intervention states using a mixed model that allows for repeated longitudinal outcomes and practice-level clustering, and is adjusted for season to deal with expected seasonal variation in HbA1c. The primary analysis will be based on the intention-to-treat (ITT) population, i.e. all eligible patients will be included. Missing outcome data will need to be imputed for the ITT analyses. This will be done using an appropriate multiple imputation method; it is anticipated that predictive mean matching will be used to impute continuous outcomes as this is able to handle non-linearity and non-normality, and logistic regression will be used for binary outcomes. Predictive variables are likely to include practice level demographics, e.g. sex, ethnic group, or baseline HbA1c. Sensitivity analyses will repeat the primary analyses using complete cases and per protocol populations.

As secondary analyses, the ITT model from the primary analysis will be fitted for the following subgroups of interest: (1) including only patients who attended education; (2) excluding patients with HbA1c < 6.5% at baseline; (3) by baseline education attendance status; (4) by patient ethnicity and age to examine the effectiveness of the Embedding Package in groups in which there is low SSME uptake; (5) by type/format of programme offered/attended.

Secondary patient-level and practice-level outcomes will be compared in a similar manner to the primary analyses, except that practice-level outcomes will not be adjusted for cluster and the psychosocial outcomes will not account for repeated measures as they will only be measured once.

Summaries of self-reported referral and attendance will be produced using appropriate descriptive statistics for the whole dataset and by pertinent subgroups, such as sex and age. Data on SSME referrals and attendance will also be compared between data sources (i.e. self-report, practice, and provider).

A full statistical analysis plan will be written and agreed by all investigators and the trial steering committee before the data are released for analysis. Statistical significance will be defined as *p*-values less than 0.05.

#### Observational follow-up

Summaries of the outcomes measured in the observational follow-up will be produced using appropriate descriptive statistics. HbA1c at 30 months will be compared with the HbA1c estimates under intervention and control conditions in the RCT using mixed regression models accounting for repeated measures on the same patients and for the practice-level clustering. Similar analyses will be conducted for the secondary outcomes, including the process outcomes which will aid understanding about why changes are, or are not, sustained.

#### Integrated ethnographic study

Qualitative data will be analysed using the Framework Method [26]. Data will be coded and organised based on an established model for evaluating intervention design: Normalisation Process Theory (NPT) [27, 28]. Findings from the ethnographic work will be triangulated with quantitative data [29] to provide an in-depth and integrated explanation of the process of implementing the Embedding Package in a variety of contexts. The adoption of an inductive and reflexive approach will ensure rapid development and integration of findings from the earlier stages, and inform the optimisation of implementing the intervention, particularly in relation to sustainability.

#### Cost-effectiveness analysis

The general framework for the analysis is to describe the costs and effects of current levels of implementation using published estimates of the cost-effectiveness of patient education programmes, then estimate the incremental costs and benefits of increased implementation. These incremental costs and benefits will be a combination of the costs of the implementation activities, the associated increase in uptake and the cost effectiveness of the patient education programmes. This framework has been applied to implementation of QOF indicators [30] and is currently being developed further by the Department of Health Policy Research Unit for the Economic Evaluation of Health and Care Interventions (EEPRU).

The cost-effectiveness analysis will take an NHS perspective and model costs and effects over the lifetime of patients (with appropriate discounting). The costs of implementation activities will be generated from within the RCT. The Embedders will record all activity and the uptake of individual embedding activities in each provider will be recorded through an activity proforma, whilst unit costs for each activity will be generated via interviews in all providers across a sample of practices. The resources identified in each interview will be costed using either budget information from the practices/CCGs or external unit costs (e.g. Unit Costs of Health and Social Care). All other costs relating to diabetes care will be generated by the Sheffield T2DM policy model [31, 32] which will have its data sources updated through literature review and identification of the most recent unit costs.

The effects of the implementation activities will be measured in terms of quality adjusted life years (QALYs) estimated using the Sheffield T2DM policy model. The model will generate the QALYs via changes in HbA1c associated with SSME. Changes in HbA1c will be estimated in two ways. The primary analysis will be based on individual patient data from this RCT (as described above), whilst a secondary analysis will use published estimates of the effectiveness of SSME generated from a meta-analysis of RCT data. The central estimate of the incremental cost-effectiveness ratio of the Embedding Package will be presented, together with probabilistic estimates of cost-effectiveness represented in a cost-effectiveness acceptability curve. Value of information analysis will be undertaken using the Sheffield Accelerated Value of Information tool [33]. Deterministic sensitivity analysis will also be undertaken to explore the effects of uncertainties that cannot be adequately represented probabilistically; for example, the length of effect of the package, uptake rate without the package, and the mix of alternative education programmes to which patients are referred. Methods and results will be reported in line with Consolidated Health Economic Evaluation Reporting Standards (CHEERS) recommendations [34].

### Sample size

In the feasibility study an average cluster size of 460 was found across the six practices included (range 118–824) (Davies et al. Increasing uptake of self-management education programmes for type 2 diabetes in a multi-ethnic primary care setting: A feasibility study. 2019. In preparation). Recruiting 58 practices (29 to each randomisation point) will give over 90% power to detect a difference in HbA1c of 0.1% assuming an SD of 1.5%, based on United Kingdom Prospective Diabetes Study (UKPDS) [35]. There will be 80% power to detect a difference as small as 0.062%. This assumes an intra-cluster correlation coefficient (ICC) of 0.05, one baseline HbA1c measurement with one more HbA1c measurement at each randomisation point. This calculation does not take account of potential variation in cluster size, as methodological work has shown that the power of studies of a stepped wedge type design is robust to variation in cluster size [36]. The sensitivity of this calculation to changes in the average cluster size was assessed. As long as the average cluster size remains larger than 174 there will be 80% power to detect a 0.1% difference. To allow for potential cluster drop out of 10% the target will be to recruit 66 practices in total.

Return of a completed questionnaire booklet (self-report questionnaire and/or consent to link data form) will be recorded as consent to participate in the study. Additionally stakeholders and patients consenting to ethnographic interviews and observations will be recorded as participants. It is anticipated a minimum of 2050 participants will be recruited (2000 via questionnaire and 50 via interview/observation).

The integrated ethnographic study will be conducted within a sub-sample of approximately 12 of the practices and related contexts (e.g. CCGs and providers). Purposive sampling of practices will take place in the first and second steps of the RCT to ensure a representative sample [22]. A subset of 12 practices will provide data for the cost-effectiveness analysis.

### Dissemination

The team have a strong track record in generalising, disseminating and implementing research findings. Locally, results will be shared with primary and secondary healthcare organisations, public health bodies and local authorities. The community will be informed of key findings via a multimedia campaign using high profile public venues and services, local press, and accessible social media.. Local ethnic and cultural communities will be contacted through PPI groups and specialist local media.

Nationally, results will be submitted for publication in peer-reviewed journals and to the NIHR Library and for presentation at NHS, health policy, commissioning, diabetes and nursing conferences, to achieve wide dissemination to academic and clinical communities. The researchers will link with key NHS stakeholders (The Royal College of General Practitioners, the East Midlands Academic Health Science Network, The King’s Fund, the Strategic Clinical Network, the Health Foundation, the South Asian Health Foundation, and the NIHR, particularly Applied Research Centres, and Academic Health Science Networks) specifically with implementation in mind, holding regional events for NHS stakeholders. Findings appropriate to the general public will be disseminated through public organisations, national and local diabetes, third sector, the national Healthwatch network, and Diabetes UK. In consultation with PPI groups, we will take direction for prioritising methods of sharing key findings through popular media, through public information networks, and web-based technology. Evaluation of the impact of our communications strategy will be made through analysing the number of publications covering outcomes, web page hits and other success measures.

### Programme steering committee

A Data Monitoring Committee was not set up for this study given the low risk nature of the intervention, i.e. the intervention is aiming to increase uptake to established programmes which are recommended by NICE. An independent Programme Steering Committee has been appointed and agreed by the funders.

## Discussion

This mixed methods study will assess the effectiveness, cost-effectiveness and sustainability of the Embedding Package, an intervention which aims to improve biomedical and psychosocial outcomes of people with T2DM, through increased referral to and uptake of SSME. This study is timely. In 2017 the James Lind Alliance published the top ten research priorities in Diabetes, and the question “What is the best way to encourage people with type 2 diabetes, whoever they are and wherever they live, to self-manage their condition, and how should it be delivered?” was ranked third, highlighting the importance of this issue to patients with T2DM, carers, health-care professionals, and ethnic minority groups [37]. Given the high burden of disease and the current low levels of uptake to education, this study has the potential to have a big impact on the management of T2DM within the UK.

This trial is informed by a programme of work including development of the Embedding Package based on evidence from existing studies to identify experiential practices, procedures, strategies and plans which represent best practice. A feasibility study was then conducted which gave an opportunity to pilot the intervention and refine it based on ethnographic findings and to assess the feasibility of data collection processes. The results of the pre-trial studies are described in detail elsewhere (Davies et al. Increasing uptake of self-management education programmes for type 2 diabetes in a multi-ethnic primary care setting: A feasibility study. 2019. In preparation).

The trial not only assesses the effectiveness of the intervention but also includes an observational follow up phase. During this time no active trial input will happen, and the follow-up phase will therefore capture whether the Embedding Package can change culture and practice leading to longer term improvements. The trial is also pragmatic; all people with T2DM will be included irrespective of whether they are referred to or attend education during the study period. Therefore a small change in HbA1c is expected. In a study conducted at the patient level, a minimum difference of 0.3% in Hba1c may be clinically meaningful and lead to change in practice. Here the study is powered to detect a difference of at least 0.1%. This reflects the practice-level design of the study. Planned secondary analyses will try to elicit the effectiveness of the intervention in those referred to education during the course of the study. The study uses a wait-list design, similar to a stepped wedge design but with a single step during the study. The design is efficient as each practice acts as its own control and therefore produces a significant reduction in the sample size required compared to a standard cluster randomised trial. Given components of the intervention are delivered at the level of the CCG/providers, strategies to limit contamination of control state practices will be put in place during the first step.

If the Embedding Package is found to be both effective and cost-effective, a ‘toolkit’ to enable widespread implementation will be developed and rolled-out.

## Data Availability

Not applicable.

## Abbreviations

BMI: Body mass index
CCG: Clinical Commissioning Group
DESMOND: Diabetes Education and Self-Management for On-going and Newly Diagnosed
HCP: Healthcare professional
HDL: High-density lipoprotein
ICC: Intra-cluster correlation coefficient
LDL: Low-density lipoprotein
MRC: Medical Research Council
NHS: National Health Service
NICE: National Institute for Health and Care Excellence
NPT: Normalisation Process Theory
PPI: Patient and public involvement
QOF: Quality and Outcomes Framework
RCT: Randomised controlled trial
SSME: Structured self-management education
T2DM: Type 2 diabetes mellitus
UK: United Kingdom
X-PERT: X-PERT Health Diabetes Education Programme
